# microRNA-3646 serves as a diagnostic marker and mediates the inflammatory response induced by acute coronary syndrome

**DOI:** 10.1080/21655979.2021.1967066

**Published:** 2021-09-14

**Authors:** Jinming Yu, Yongmei Li, Deguo Leng, Cheng Cao, Yongzhi Yu, Yijuan Wang

**Affiliations:** a Department of Clinical Laboratory, Zibo Municipal Hospital, Zibo, Shandong, China; b Department of Cardiology, Zibo Municipal Hospital, Zibo, Shandong, China

**Keywords:** miR-3646, acute coronary syndrome, inflammatory response

## Abstract

Acute coronary syndrome (ACS) is one of the main syndromes of coronary artery disease with high mortality. The identification of biomarkers associated with disease occurrence and progression could improve early detection and risk prediction. This study was aimed to reveal the clinical significance and function of miR-3646 in ACS.

The expression of miR-3646 was evaluated in ACS patients, healthy volunteers, and non-ACS patients and estimated the clinical significance of miR-3646. The ACS modeling rats were also established in this study to explore the potential mechanism underlying the function of miR-3646. miR-3646 was upregulated in ACS patients compared with healthy volunteers and non-ACS patients. The expression of miR-3646 was positively correlated with the severity and progression of ACS patients and could discriminate ACS patients from healthy volunteers and non-ACS patients. The knockdown of miR-3646 could reverse the inflammatory response induced by ACS.miR-3646 serves as a diagnostic biomarker for ACS. The knockdown of miR-3646 could alleviate ACS by reversing inflammatory response. These results provide a potential therapeutic target of ACS.

## Introduction

Coronary artery disease (CAD) is one of the most prevalent cardiovascular diseases, which accounts for a large proportion of morbidity and mortality. Myocardial infarction, acute coronary syndrome (ACS), and chronic coronary heart disease are the major manifestation of CAD [1]. ACS usually results from myocardial ischemia after unstable coronary atherosclerotic plaque formation [2]. The clinical presentations include acute myocardial infarction and unstable angina. The assessment of symptoms, ischemia changes in electrocardiogram (ECG), and troponin are conventional means for the diagnosis of ACS [3]. While the changes in ECG were always sensitive and easily affected by the patient’s condition, such as left bundle branch block and chronic myocardial infarction [4]. There is still a research gap in the approach to the early detection and development prediction of ACS. Therefore, the identification of early diagnostic biomarkers of ACS is of urgent need.

In clinical diagnosis, LDH, a key enzyme released after cell membrane damage, is one of the biochemical markers except for troponin. Recently, numerous studies have demonstrated that the expression levels of microRNAs (miRNAs) had varying degrees of dysregulation in ACS. miRNAs are encoding RNAs approximate to 22–28 nucleotides in length and possess the function of suppressing target gene expression through binding with mRNA at specific sequences [5]. In the past decades, miRNAs have been identified as biomarkers to monitor the occurrence and development of various human diseases, such as cardiovascular disease and human cancers [6,7]. Several miRNAs have been revealed to play roles in the development of ACS. For instance, miR-941 was identified as a promising biomarker for the diagnosis of ACS [8]. miR-206 suppresses the progression of CAD and regulates the expression of VEGF [9]. The increased expression level of miR-122-5p might predict the severity of coronary lesions [10]. The overexpression of miR-335-5p was illustrated to suppress the atherosclerotic vulnerable plaque formation and the innate immune response of macrophage in ACS [11]. Barraclogh et al. reported that miRNAs were also involved in the anti-tumor effect of colchicine, as their expression levels were regulated by colchicine and these miRNAs mediate the treatment efficacy of colchicine [12]. miR-3646 has been identified as an upregulated miRNA in ACS implying its potential clinical significance in ACS [13]. Moreover, miR-3646 has been demonstrated to promote vascular inflammation and the proliferation of vascular smooth muscle cells in the coronary artery [14]. Therefore, it is speculated that miR-3646 could serve as a biomarker for the diagnosis of ACS.

The purpose of this study was to investigate the expression level of miR-3646 in ACS and assess the clinical significance of miR-3646. Additionally, it was also aimed to reveal the mechanism underlying the function of miR-3646.

## Materials and methods

### Patients and samples

This study complied with the Declaration of Helsinki and had obtained approval from the Ethical Committee of Zibo Municipal Hospital. A total of 72 ACS (29 unstable angina (UA), 22 non-ST elevation ACS, and 21 ST-segment elevation myocardial infarction (STEMI)) patients and 50 healthy volunteers were included in this study from 2017 to 2019. The inclusion criteria were: a) age over 18 years old; b) the first attack of chest discomfort occurred within 2 months; c) The lumen diameter of the left main coronary artery, left anterior descending branch, left circumflex branch, or right coronary artery was with stenosis over 50%. Patients suffered from uncontrolled infectious diseases, autoimmune disease, malignancy, or pregnancy were excluded. Another 63 patients with negative findings by coronary angiography were also included as a negative control to evaluate whether the dysregulation of miR-3646 was induced by ACS. The clinicopathological features were summarized in Table 1. A total of 2 mL venous blood samples was collected from every participator and mixed with EDTA as an anticoagulant. The collected samples were centrifuged at 2000 rpm for 10 min to obtain serum samples. The serum samples were stored at −80°C until the following analysis.

**Table 1.** The clinical features of study subjects

	Healthy	ACS	Non-ACS
Age	61.68 ± 2.13	62.26 ± 3.40	62.02 ± 2.52
Sex (M/F)	28/22	39/33	35/28
TC (mmol/L)	3.79 ± 0.72	4.42 ± 0.74***/^###^	3.71 ± 0.32
HDL (mmol/L)	1.31 ± 0.57	1.25 ± 0.39	1.35 ± 0.29
LDL (mmol/L)	2.45 ± 0.76	2.90 ± 0.80***/^###^	2.48 ± 0.66
GLU (mmol/L)	5.62 ± 0.48	5.95 ± 0.62***/^##^	5.68 ± 0.50
TG (mmol/L)	1.70 ± 0.39	1.76 ± 0.55	1.68 ± 0.51
Type (UA/NSTEM/STEMI)	-	29/22/21	-
Gensini score	-	61.59 ± 11.22	-

ACS: acute coronary syndrome; TC: total cholesterol; HDL: high density lipoprotein; LDL: low density lipoprotein; GLU: blood glucose; TG: triglycerides; UA: unstable angina; NSTEM: non-ST elevation ACS; STEMII: ST-segment elevation myocardial infarction. ***P < 0.001 compared with the healthy group; ^##^*P* < 0.01, ^###^*P* < 0.001 compared with the ACS group

### RNA extraction and PCR analysis

The assessment of miR-3646 was conducted with the help of PCR according to previous studies [15]. Total RNA was extracted from collected samples with RNAqueous Kit (Life Technologies, USA) and generated cDNA with the SuperScript III Reverse Transcriptase (Invitrogen, USA) according to the manufacturer’s instructions. The expression of miR-3646 was evaluated by the 7300 real-time PCR system with the SYBR Green I Master Mix kit (Invitrogen, USA) and calculated by the 2^−ΔΔCt^ method normalized to U6. The primer sequences of miR-3646 were: forward 5ʹ-CCCCAAAATGAAATGAGCC-3ʹ, reverse 5ʹ- CAGTGCGTGTCGTGGAGT-3ʹ, and the sequences of U6 were: forward 5ʹ- CGCAAGGATGACACG-3ʹ, reverse 5ʹ- GAGCAGGCTGGAGAA-3ʹ. The PCR was conducted at following conditions: denature at 95°C for 5 min, 35 cycles of 95°C for 30 s, then 72°C for 30 s, and a final extension at 72°C for 10 min.

### ACS animal modeling

The establishment of ACS animal models was conducted according to previous studies [16]. Male Sprague-Dawley rats (weighted 220–250 g) were used to establish the ACS animal model by the coronary artery ligation. Rats were anesthetized with 1% pentobarbital sodium injection and the 12-lead ECG was recorded. During the establishment, the rats were intubated and connected to an animal ventilator. The skin and subcutaneous tissues around the third and fourth rib were cut up and separated the pectoralis major muscle and the serratus anterior muscle. The left atrial appendage was separated from the pulmonary conus by cutting up the third costal cartilage near the sternal margin. The left coronary artery was punctured by the 6–0 ophthalmic noninvasive suture needle and the small bundle of myocardial ligation and ligation sites was observed. The weakened myocardial movement, increasing ST segments, and lead or chest leads elevated more than 0.2 mV with corresponding lead changes, indicating the successful ligation.

The chest was closed layer by layer after checking bleeding, and the rats recovered spontaneous respiration. Penicillium was injected after surgery to avoid infection. After 8 weeks of the establishment of the ACS model, all rats were subjected to ECG and hemodynamic measurement to assess whether the modeling was successful.

### Grouping

The rats were divided into three groups randomly including the sham group, ACS group, and ACS+ anti-miR-3646 group with six rats of each. The ACS+ anti-miR-3646 group was established by injecting miR-3646 interference plasmids via the tail vein in ACS modeling rats. After corresponding treatment, the blood samples were collected and stored at −80°C for the following analysis.

### Enzyme-linked immunosorbent assay (ELISA)

The concentrations of pro-inflammatory cytokines were detected by ELISA to evaluate the inflammatory reaction. The detection was conducted with the ELISA kit (Quantikine Immunoassay, R&D Systems, USA) according to the manufacturer’s instructions [17].

### Statistical analysis

All data were represented as mean value ± SD. obtained from at least triplicate experiments. The difference between the two groups was evaluated by Student’s t-test followed by the Turkey post-hoc, and the difference between multiple groups was estimated by one-way ANOVA followed by the Turkey post-hoc. The association between miR-3646 expression and clinicopathological features of patients was assessed by Chi-square test and Pearson’s correlation analysis. *P* < 0.05 was considered to be statistically significant.

## Results

The reported dysregulation of miR-3646 in the previous study implied the potential clinical significance of miR-3646 in ACS. To evaluate the clinical significance of miR-3646 in ACS and disclose the potential mechanism, miR-3646 was investigated in ACS patients and ACS animal models.

### The clinical features of study subjects

The healthy volunteers were composed of 28 males and 22 females with an average age of 61.68 ± 2.13 years old. The ACS patients included 39 males and 33 females with an average age of 62.26 ± 3.40 years old. There were 35 males and 28 females in the non-ACS groups with an average age of 62.02 ± 2.52. The age and gender composition showed no significant difference between the three groups (*P* > 0.05, Table 1). The concentration of triglycerides (TG) between the three groups was not significantly different (*P* > 0.05, Table 1). The total cholesterol (TC), low density lipoprotein (LDL), and blood glucose (GLU) levels were significantly increased in ACS patients compared with healthy volunteers and non-ACS patients (*P* < 0.01, *P* < 0.001, Table 1).

### The expression and diagnostic value of miR-3646 in ACS patients

**Figure 1.**
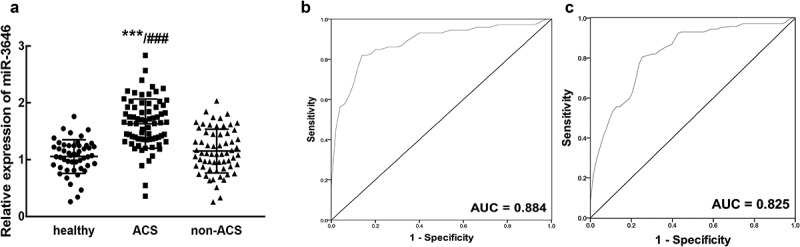
The expression of miR-3646 and its diagnostic value in ACS patients. A. miR-3646 was significantly upregulated in the serum of ACS patients compared with that in healthy volunteers and non-ACS patients. ***P < 0.001 relative to healthy volunteers; ^###^*P* < 0.001 relative to non-ACS patients. B. miR-3646 could distinguish ACS patients from healthy volunteers with an AUC of 0.884. C. miR-3646 could distinguish ACS patients from non-ACS patients with the AUC of 0.825

**Figure 2.**
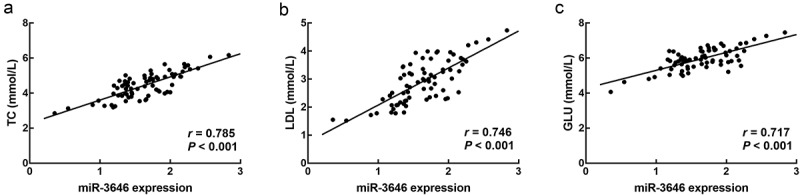
The correlation of miR-3646 with the TC (a), LDL (b), and GLU (c) levels of ACS patients. miR-3646 showed significantly positive correlation with the TC (*r* = 0.785), LDL (*r* = 0.746), and GLU (*r* = 0.717) levels of ACS patients

In the serum of ACS patients, miR-3646 was found to significantly upregulate compared with that of the healthy volunteers and non-ACS patients (*P* < 0.001, Figure 1(a)). Moreover, from the results of ROC curve, it was found that miR-3646 could distinguish ACS patients from healthy volunteers (AUC = 0.884, specificity = 0.880, sensitivity = 0.819, Figure 1(b)) and non-ACS patients (AUC = 0.825, specificity = 0.746, sensitivity = 0.819, Figure 1(c)) with high specificity and sensitivity. Based on the average expression of miR-3646 in ACS patients, 72 patients were divided into a low expression group (n = 34) and a high expression group (n = 38). The upregulation of miR-3646 in ACS patients showed significant association with the TC (*P* = 0.030), LDL (*P* = 0.008), GLU (*P* = 0.007) levels, and the Gensini scores (*P* = 0.021) of ACS patients (Table 2). Furthermore, the TC (Figure 2(a)), LDL (Figure 2(b)) and GLU (Figure 2(c)) levels of ACS patients showed positive correlations with the expression of miR-3646 with the correlation coefficients of 0.785, 0.746, and 0.717, respectively (*P* < 0.001).

**Table 2.** Association between miR-3646 expression and clinical features of ACS patients

	Total (72)	miR-3646 expression		*P* value
		Low (34)	High (38)	
Age (years)				0.637
≤ 60	36	18	18	
> 60	36	16	20	
sex				0.782
Male	39	19	20	
Female	33	15	18	
TC (mmol/L)				0.030
≤ 4.5	39	23	16	
> 4.5	33	11	22	
HDL (mmol/L)				0.260
≤ 1.25	31	17	14	
> 1.25	41	17	24	
LDL (mmol/L)				0.008
≤ 3.0	39	24	15	
> 3.0	33	10	23	
GLU (mmol/L)				0.007
< 6.0	41	25	16	
≥ 6.0	31	9	22	
TG (mmol/L)				0.803
< 1.7	35	16	19	
≥ 1.7	37	18	19	
Type				0.103
UA	29	11	18	
NSTEM	21	14	7	
STEMI	22	9	13	
Gensini score				0.021
< 60	30	19	11	
≥ 60	42	15	27	

ACS: acute coronary syndrome; TC: total cholesterol; HDL: high density lipoprotein; LDL: low density lipoprotein; GLU: blood glucose; TG: triglycerides; UA: unstable angina; NSTEM: non-ST elevation ACS; STEMII: ST-segment elevation myocardial infarction.

### The function of miR-3646 in ACS modeling rats

**Figure 3.**
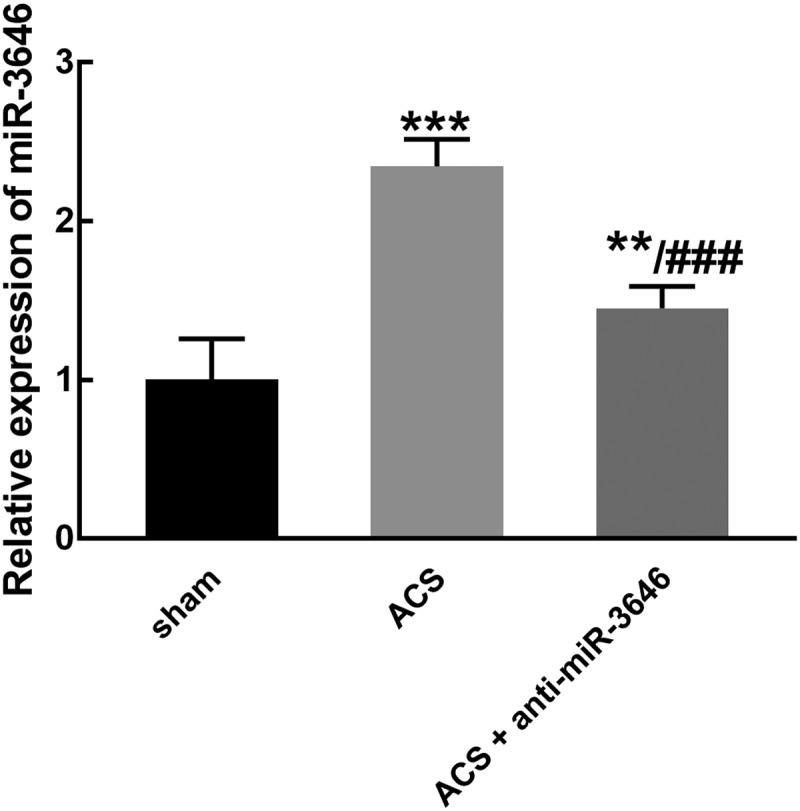
The expression of miR-3646 in ACS modeling rats was obtained from six rats in each group. miR-3646 was significantly upregulated in ACS modeling rats, which was reversed by the injection of miR-3646 interference plasmid. **P < 0.01, ***P < 0.001 relative to the sham group; ^###^*P* < 0.001 relative to the ACS group

miR-3646 was significantly higher in ACS modeling rats than that in the sham group, while in the anti-miR-3646 group, the upregulation of miR-3646 was reversed and it was still significantly higher than that in the sham group (*P* < 0.01, *P* < 0.001, Figure 3).

**Figure 4.**
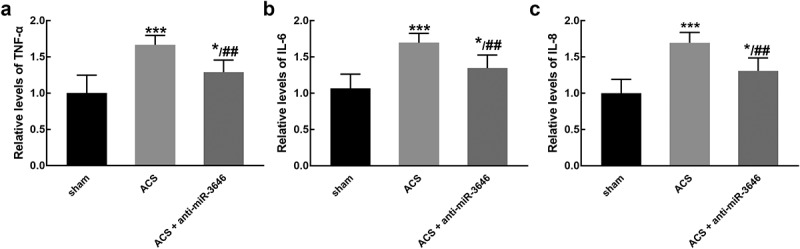
The effect of miR-3646 on the relative levels of pro-inflammatory cytokines was studied in six rats of each group. ACS induced the increasing levels of TNF-α (a), IL-6 (b), and IL-8 (c), which was reversed by the knockdown of miR-3646. *P < 0.05, ***P < 0.001 relative to the sham group; ^##^*P* < 0.01 relative to the ACS group

ACS induced a significant increase in the levels of pro-inflammation cytokines, including TNF-α (Figure 4(a)), IL-6 (Figure 4(b)), and IL-8 (Figure 4(c)), indicating the occurrence of inflammatory reaction (*P* < 0.001). While the knockdown of miR-3646 reversed the increasing levels of TNF-α (Figure 4(a)), IL-6 (Figure 4(b)), and IL-8 (Figure (4c)) induced by ACS, suggesting miR-3646 alleviated the inflammatory reaction (*P* < 0.05, *P* < 0.01).

**Figure 5.**
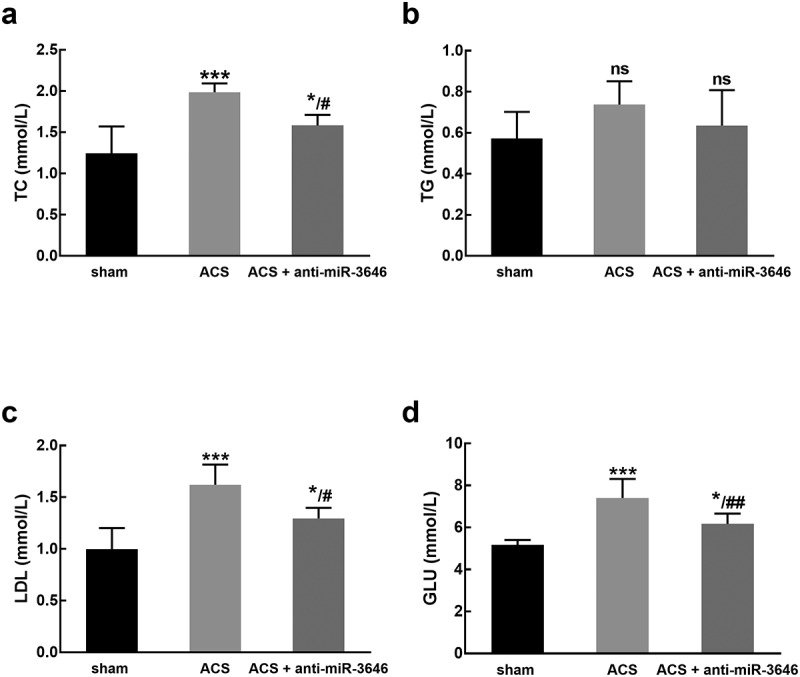
The effect of miR-3646 on the TC (a), TG (b), LDL (c), and GLU (d) of rats (six rats of each group). ACS induced a significant increase in the levels of TC, LDL, and GLU, while no significant changes were observed in the level of TG. *P < 0.05, ***P < 0.001 compared with the sham group; #*P* < 0.05, ##*P* < 0.01 compared with the ACS group

Additionally, the TC, TG, LDL, GLU levels of experimental animals were detected. It was found that ACS modeling led to the increasing levels of TC (Figure 5(a)), LDL (Figure 5(b)), and GLU (Figure 5(c)), which was attenuated by the knockdown of miR-3646 (*P* < 0.05, *P* < 0.01, *P* < 0.001, Table 3).

**Table 3.** The corresponding features of rats in each group

	Sham	ACS	ACS + anti-miR-3646
TC (mmol/L)	1.25 ± 0.33	1.99 ± 0.11***	1.58 ± 0.13*/^#^
TG (mmol/L)	0.57 ± 0.13	0.74 ± 0.11	0.64 ± 0.17
LDL (mmol/L)	1.00 ± 0.20	1.62 ± 0.20***	1.30 ± 0.10*/^#^
GLU (mmol/L)	5.17 ± 0.24	7.41 ± 0.91***	6.17 ± 0.50*/^##^

ACS: acute coronary syndrome; TC: total cholesterol; LDL: low density lipoprotein; GLU: blood glucose; TG: triglycerides.

*P < 0.05, ***P < 0.001 compared with the sham group; ^#^*P* < 0.05, ^##^*P* < 0.01 compared with the ACS group.

## Discussion

Nowadays, the functional role of miRNAs in various human diseases attracted special attention. A number of miRNAs have been identified as a biomarker for the pathogenesis and progression of human diseases, such as malignant tumor, coronary artery disease, and Alzheimer’s disease [18–26] miR-3646 has been considered as a regulator in breast cancer that promotes cell proliferation, migration, and invasion and enhance the resistance to paclitaxel [27,28]. The dysregulation of miR-3646 was also demonstrated in ACS in a previous study, and miR-3646 was revealed to enhance vascular inflammation and mediate the proliferation and migration of vascular smooth muscle cells, which are associated with the progression of coronary artery disease [13,14]. However, the specific role of miR-3646 in ACS was still unclear.

Numerous molecules have been reported to possess diagnostic value in ACS. For example, miR-587 showed a close association with the severity of ACS patients and was considered to be a potential biomarker for the diagnosis and prognosis of ACS [29]. miR-145 was disclosed to participate in the dysfunction of endothelial cells and differentiate ACS patients from healthy controls [30]. In this study, miR-3646 was found to be upregulated in ACS patients and discriminate ACS patients from healthy volunteers and non-ACS patients with high sensitivity and specificity. Chest pain radiating to the angle of the jaw or left shoulder is the most common symptom of ACS. The diagnosis of ACS usually depends on the ECG and troponin assessment, which are easily affected by the environmental factors or the status of patients [31,32]. The identification of a novel diagnostic biomarker, miR-3646, could improve the early detection of ACS and the therapy of patients. Meanwhile, miR-3646 was also found to be positively correlated with the TC, LDL, and GLU levels of patients, which are closely associated with the occurrence and development of ACS [33–36]. miR-3646 was also observed to exert significant association with the Gensini scores of ACS patients, which represents the severity and disease development of ACS [37]. Consistently, in the ACS modeling rats, the upregulation of miR-3646 was also observed with a positive relationship with the TC, LDL, and GLU levels of rats. These results revealed the clinical significance of miR-3646 in the diagnosis and progression of ACS.

Further, the potential mechanism underlying the function of miR-3646 was explored. The inflammatory response is the main symptom and the key process in the pathogenesis of ACS [38]. The mediation of miR-3646 on vascular inflammation was suggested to be the potential mechanism underlying the involvement of miR-3646 in coronary artery disease and vascular smooth muscle cell proliferation [14]. In previous study, it has been reported that miR-330 could regulate the levels of pro-inflammation cytokines and therefore mediated the formation of atherosclerotic plaques and the proliferation of vascular endothelial cells [39]. The increasing levels of pro-inflammation cytokines were found in the ACS modeling rats, and it was reversed by the knockdown of miR-3646, indicating regulating inflammatory response might be the potential mechanism by which miR-3646 participated in the progression of ACS.

The molecular mechanism investigation was lacked in the present study, which is a limitation of this study. miR-3646 was demonstrated to directly target RHOH to promote vascular inflammation in coronary artery disease [14]. RHOH might be the downstream target of miR-3646 during its function in ACS, which needs further validation in the future study. The present study only focused on the single effect and function of miR3646 in the diagnosis and disease development of ACS. The crossed effects between miR-3646 and other factors, such as patients’ clinical features, need to be dug out in further investigations. Additionally, this study only focused on the function and significance of miR-3646 in ACS, which neglected the role of other promising miRNAs with abnormal expression. Further investigations are needed to leak out effective biomarkers for the progression of ACS.

## Conclusion

Taken together, upregulated miR-3646 could distinguish ACS patients from healthy volunteers and non-ACS patients. miR-3646 showed significant association with the key clinical features of ACS patients, which are closely associated with the severity of ACS patients, indicating its involvement in the development of ACS. miR-3646 showed a significant inhibitory effect on the inflammatory response induced by ACS, which was speculated as the mechanism underlying the function of miR-3646.
